# Machine learning-based discovery of GW3965 as a therapeutic compound against invasive emm92-type group A Streptococcus

**DOI:** 10.21203/rs.3.rs-8779898/v1

**Published:** 2026-02-18

**Authors:** Lillie M. Powell, Megan Grund, Wenxian Shi, Soo Jeon Choi, Keilly Zelaya, Sharan Bobbala, Mariette Barbier, P. Rocco LaSala, Slawomir Lukomski

**Affiliations:** West Virginia University; West Virginia University School of Medicine; Massachusetts Institute of Technology; West Virginia University; West Virginia University; West Virginia University; West Virginia University; West Virginia University; West Virginia University

**Keywords:** group A Streptococcus, antimicrobial resistance, machine learning, artificial intelligence, GW3965

## Abstract

A multi-drug resistant *emm92*-type strain of group A *Streptococcus* (GAS) has emerged as an important causative agent of invasive infections – particularly affecting people who inject drugs - in the United States. To curtail this developing threat, we aimed to identify and repurpose FDA-investigated compounds as antimicrobials. To identify growth-inhibiting compounds, a machine learning-based model was trained on the *emm92*-iGAS growth response to 2,560 bioactive compounds. The model was used to screen a 6,111-compound library of FDA-evaluated drugs *in silico*. The predicted GW3965 compound experimentally exhibited a 99% reduction in iGAS survival at an MIC of 6.25 μM. Treatment with GW3965 aided complete wound closure in a human skin equivalent model, and significantly decreased lesion size and reduced bacterial burden in a mouse model of skin and soft tissue infection. Application of a machine learning model expedited the discovery of GW3965 as a therapeutic for iGAS skin and soft tissue infections.

## Introduction

*Streptococcus pyogenes* or group A *Streptococcus* (GAS) is a gram-positive bacterial pathogen responsible for ~ 730 million infections per year globally, which range in severity from superficial pharyngitis and pyoderma to invasive infections^1^. Presentation of invasive GAS (iGAS) infections include skin and soft tissue infections (SSTI), necrotizing fasciitis, streptococcal toxic shock syndrome, and bacteremia^2^. The number of iGAS infections have been on the rise in the U.S., doubling in number of cases and deaths since 2009^3^. The standard treatment for iGAS disease is combination therapy with a β-lactam and clindamycin^4^. However, per the 2019 CDC antibiotic resistance threats report^3^ and the WHO’s 2024 priority pathogens list^5^, iGAS resistance to erythromycin and clindamycin is a growing concern^6–9^. In the U.S., the MLS_B_ phenotype of resistance to 3 classes of antibiotics, macrolides, lincosamides and streptogramin B, is primarily attributed to the acquisition of *erm* (erythromycin resistance methylase) genes, which encode methyltransferase enzymes^6,10,11^.

In the absence of an approved GAS vaccine, tracking the predominant and emerging iGAS *emm*-types is critical to determine shifts in epidemiological trends. Surveillance of iGAS isolates by the CDC’s Active Bacterial Core at 10 sites across the U.S. shows that the *emm1*, *emm12*, *emm28*, and *emm3* strains continue to cause the majority of invasive infections^8,12,13^. However, CDC and local surveillance reports support a significant and growing presence of MLS_B_, aminoglycoside and tetracycline-resistant isolates between 2010–2022, of which the *emm92* strain was a predominant cause of infection^9,13–17^. The *emm92* strain has been majorly associated with the at-risk intravenous drug use (IVDU) population^6,9,10,13,18,19^. This trend is particularly evident in the rural West Virginia population, where from January 2020–2023 46% of infections were caused by a multi-drug resistant *emm92* strain, among which 62% were associated with a patient history of IVDU^15,16^. Therefore, the *emm92* strain serves as a model organism for evaluation of compound efficacy against resistant iGAS infections due to recent emergence and carriage of MLS_B_, aminoglycoside, and tetracycline resistance.

Our goal here was to identify novel antimicrobials effective against the GAS pathogen responsible for invasive skin and soft tissue infections. We developed a machine learning model trained to predict compounds with inhibitory activity against *emm92* iGAS, which serves as a model multi-drug resistant strain, through application of growth inhibitory responses to a diverse compound library, similar to as discussed previously^20,21^. The trained neural network was then used for *in silico* screening of FDA-evaluated small compounds. Investigation of FDA-evaluated compounds provided the advantage of identifying drugs with known safety profiles that could be re-purposed as antimicrobials. Through this approach, we identified GW3965, as an antimicrobial for iGAS infections. GW3965 promoted wound closure in a human skin equivalent *in vitro* model and reduced disease severity in a murine model of iGAS skin and soft tissue infection. This study employed machine learning and AI to expedite antimicrobial discovery to combat the emerging threat of erythromycin and clindamycin resistant iGAS.

## Results

### Generation of an inhibitory compound dataset for training of the DML-AI model

**Figure 1:**
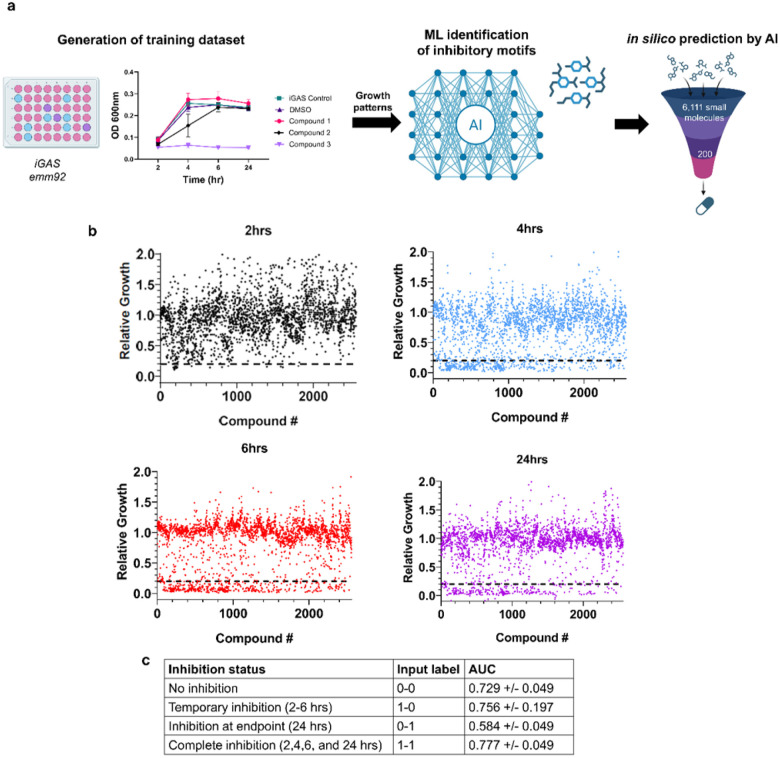
Development of a machine learning model for prediction and identification of inhibitory compounds. **a)** Workflow for model development and compound prediction. To generate a training dataset a small compound library was screened against *emm92*-15 WV-iGAS. **b)** Relative growth of each compound to DMSO control at indicated timepoints. The OD_600nm_ measurement of growth at the 2-, 4-, 6-, and 24-hour timepoints was used to determine the percentage of growth inhibition relative to the DMSO culture control. Dotted line represents 80% growth inhibition according to turbidity, where a value of 1 represents normal growth. **c)** Patterns of growth inhibition used to train the machine learning model. Training supported the model’s capacity to identify motifs associated with antimicrobial activity based on identification of inhibitory structures and substructures. Some images created with BioRender.com.

To identify novel antimicrobials against the *emm92-*15 iGAS strain using deep machine learning, we generated a training dataset of inhibitory and non-inhibitory compounds by screening a library of bioreactive and natural small compounds containing metabolites, medicinal compounds, and antimicrobials, including known antibiotics (Fig. 1a). Compounds were classified as inhibitory if culture turbidity (OD_600nm_) was ≤ 20% of the medium-drug-solvent control (THY broth, 2.5% DMSO), labeled as relative growth. Of the 2,560 compounds screened, we identified 22 inhibitory compounds after a 2-hour incubation, 255 after 4-hours, 274 after 6-hours, and 191 after 24-hours (Fig. 1b). Most of the inhibitory compounds were functionally categorized as antibacterial, followed by antineoplastic, antifungal, and anti-inflammatory, as well as antidepressants and antihypertensives (Tables S1). Thus, we generated a diverse training dataset containing known antibiotics and novel inhibitory compounds.

Using this dataset, we trained a deep machine learning model to establish common structures and substructures associated with growth inhibition, with the goal of training the model to reliably predict inhibitory activity of a compound after 24-hours of incubation. We found our model could reasonably predict inhibitory activity at the 24-hour timepoint (AUC = 0.776 +/− 0.052). To improve model prediction, we further classified inhibition activity into four distinct profiles by incorporating data for the intermediate time points of 2, 4, and 6 hours. Accordingly, compounds were categorized into four different kinetic growth inhibition profiles: (i) no inhibition, (ii) temporary inhibition (2–6 hours), (iii) delayed inhibition (inhibitory at 24 hours), or (iv) complete inhibition across all timepoints (Fig. 1c). These 4 categories were employed as growth profiles can allude to a compound’s mechanism of action, where complete inhibition is indicative of bactericidal activity, whereas delayed inhibition can occur from possible cell division arrest. With this approach, we found our model could predict well compounds that exhibited complete (AUC = 0.777 +/− 0.049), temporary (0.756 +/− 0.197), or no inhibition (AUC = 0.729 +/− 0.049). Using the multi-class predictions, we also observe a slight improvement in predicting 24-hour inhibition activity. Specifically, we aggregate the predicted probabilities of delayed and complete inhibition as the score for inhibition at 24 hours, and those of temporary and no inhibition as the score for no inhibition at 24 hours. This yields an AUC of 0.783 ± 0.052. Based on this data, we hypothesize that, rather than focusing solely on the inhibition outcome at 24 hours, incorporating intermediate inhibition states provided additional supervisory signals to help the model learn how molecular structures relate to dynamic inhibition behaviors.

### In silico prediction and validation of montelukast and GW3965 antimicrobial activity

A key challenge in drug development is obtaining FDA approval for clinical trials; thus, we sought to identify FDA-evaluated drugs suitable for repurposing against iGAS infections. Therefore, the trained model was applied for *in silico* screening of inhibitory compounds from a Drug Repurposing Hub of 6,111 small molecules at various stages of FDA investigation for human diseases^22,23^ (Fig. 1a). Structural information generated by the machine learning model was used to predict iGAS growth inhibition profiles *in silico* with outputs between 0 (no inhibition) and 1 (complete inhibition) to select targets. To identify the most promising compounds, we focused on the 200 compounds with the highest predicted scores. After elimination of the known antimicrobial drugs (scores above 0.712), compounds with scores ranging from 0.15 to 0.711 were considered for further evaluation. The top list of compounds was then categorized by functionality and mechanism of action. Nine representatives with various known mechanisms of action and scores ranging from 0.565–0.7 were then selected for further validation and characterization, including: montelukast sodium salt, GSK461364, AMG510, siponimod, ABT-263, miltefosine, CFTR inhibitor-172, GW3965 HCl, and probucol (Table 1). We determined minimum inhibitory concentrations (MIC) for each predicted compound by measuring bacterial growth, using two-fold drug dilutions down from 50 μM (Table 1). Montelukast sodium salt and GW3965 HCl had the best drug potency with inhibitory efficacy of 6.25 μM MIC. Miltefosine inhibited growth at a 25 μM concentration while the remaining compounds had an MIC of > 50 μM (Fig. S1a-i, Table 1). Taken together, we selected montelukast and GW3965 as the top candidates for further evaluation (Fig. 2a-b).

We next assessed the bacteriostatic or bactericidal effect of montelukast and GW3965 on iGAS survival with a 6.25 μM concentration. There was not a significant difference in viable bacteria between the media and vehicle controls. Treatment with montelukast lead to a 1-log reduction in viable bacteria corresponding to a 76% decrease in survival, which is indicative of bacteriostatic activity (Fig. 2a, bottom). In contrast, GW3965 reduced viable bacteria by 3-logs with growth inhibition of 99% (Fig. 2b, bottom), which is indicative of bactericidal action^24^. While both montelukast and GW3965 significantly inhibited bacterial growth, montelukast is marked with a black box warning for neuropsychiatric events and has mixed safety data^25–27^; thus, we focused on GW3965 for further development.

### GW3965 inhibits GAS growth in vitro

**Figure 3:**
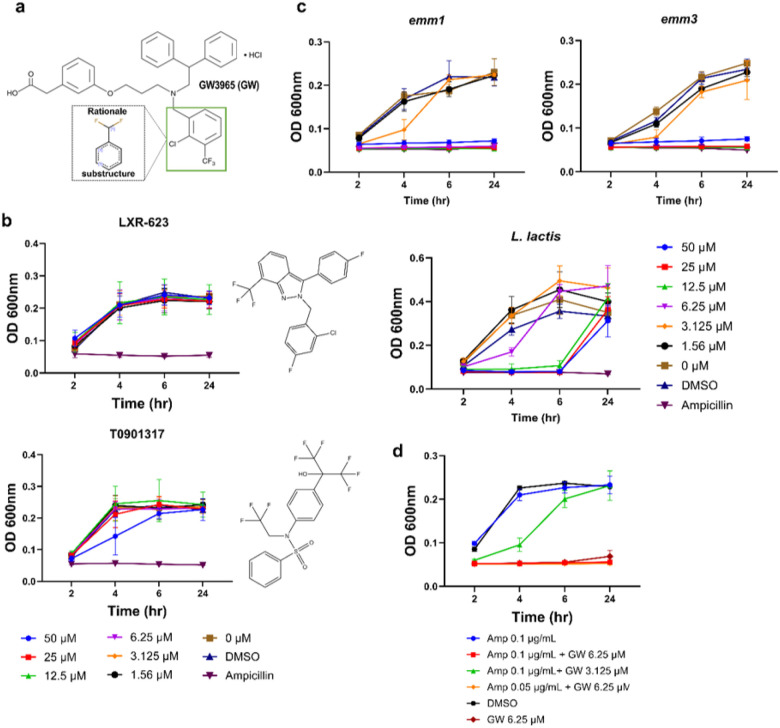
Analysis of GW3965 active group, antimicrobial strain effect, and synergy. **a)**GW3965 (GW) chemical structural interpretability and prediction. Using the Chemprop interpret module, neural networks were trained to predict inhibitory structures and substructures based on atomic and bond features represented in the chemical structural formulas called SMILES. This training takes into account local chemistry and the relationship between neighboring bonds. The predicted active group of the GW3965 molecule was the chlorobenzene ring sub-group. **b)** Growth curves of the *emm92* strain with liver X receptor agonists. LXR-623 and T0901317 were tested for antimicrobial activity against the WV-iGAS *emm92*-15 strain with a 1.56–50 μM concentration range. Structures adapted from APExBio. **c)** GW3965 activity against different iGAS strains and the probiotic *Lactococcus lactis*. The antimicrobial activity of GW3965 was assessed for the *emm1, emm3*, and*L. lactis* strains at a 1.56–60 μM concentration range. **d)**Checkerboard assay for synergy of GW3965 (GW) and ampicillin (Amp). The *emm92*strain was grown with different combinations of GW3965 (0.78–12.5 μM) and ampicillin (0.0015–0.1 ug/mL). Data from three independent experiments is shown with ± sd. GraphPad Prism software was used for figure generation.

To further characterize GW3965, we assessed the active group of GW3965 via a Monte Carlo Tree Search. We identified the chlorobenzene ring substructure retains the highest predicted antimicrobial activity of GW3965 (Fig. 3a). For comparison, we also assessed two additional liver X receptor agonists, LXR-623 and T0901317, for growth inhibition due to shared structural features with GW3965; however, neither compound reduced bacterial growth with an up to 50 μM concentration (Fig. 3b). Since antimicrobial resistance may vary between GAS strains, we tested the growth inhibition by GW3965 on two globally predominate iGAS-M types: M1 (*emm1)* and M3 (*emm3*). We found across the iGAS strains tested (e.g., M92, M1, M3), all had an MIC of 6.25 μM for GW3965. To investigate the effect of GW3965 on normal flora we determined that GW3965 was not bactericidal towards the Gram-positive probiotic *Lactococcus lactis* (Fig. 3c).

Further, combination therapy of a β-lactam with clindamycin has been used for treatment of iGAS infections, where the additive effect of clindamycin is associated with decreased toxin production^28^. Here, we investigated whether GW3965 could act synergistically with ampicillin, as an exemplar β-lactam. Using a checkerboard assay, we did not find that the two compounds acted synergistically against the *emm92* strain (Fig. 3d), with a fractional inhibitory concentration (FIC) index (MIC for ampicillin 0.12 μg/mL, GW3965 6.25 μM) of 2, indicative of an indifferent effect.

### GW3965 treatment supports wound closure and bacterial clearance in a human skin equivalent model

**Figure 4:**
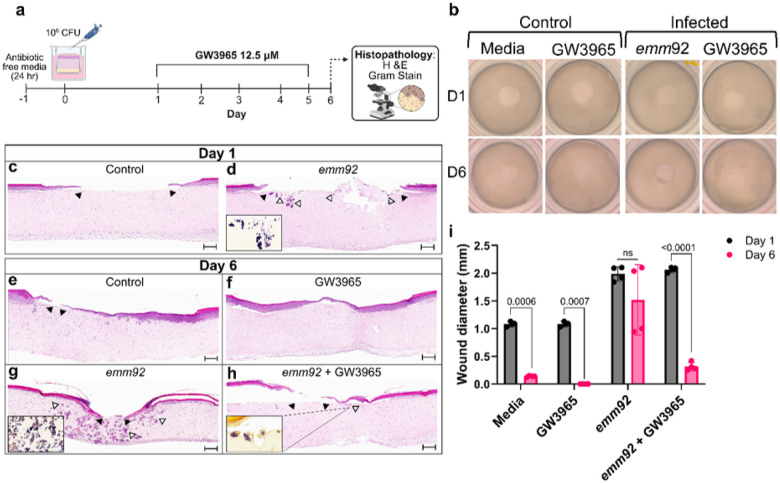
Effect of GW3965 on wound healing and bacterial burden in an *in vitro* human skin equivalent model. **a)** Experimental layout of infection, treatment, and endpoint parameters. Treatment with 12.5 μM of GW3965 through delivery in culture media was started 24 hours post-infection with the *emm92*-3 strain. Media was changed daily with GW added for 5 days. Images created with BioRender. **b)** Images of skin equivalents. Wounds were visualized at 24-hours post-infection and day 6 following treatment. **c-h)** H&E-stained tissues shown at 5x magnification; scale bar: 200 μm. Gram-stained sections are shown at 20x magnification. Black arrows indicate the progress of epidermal closure, while the white arrows show bacterial microcolony formation. **i)**Wound diameter. Wound closure was measured as the distance between the keratinocyte layer at 1.25x magnification for all samples (n=2). Diameter is shown for technical replicates of tissue sections. Comparison of lesion diameter on day 1 versus day 6 was assessed by Unpaired *t*-tests with Welch’s correction. There was no statistically significant difference between day 1 and day 6 for the *emm92* infected samples that did not receive treatment (p=0.2373). GraphPad Prism software was used for figure generation.

Human skin equivalents were utilized in an *in vitro* SSTI model to determine the effect of GW3965 on *emm92* infection. Cultured tissues were wounded by punch biopsy of the epidermal layer to mimic a skin break as a portal of entry for iGAS infection^29^. The WV-iGAS *emm92*-3 strain was used for infection studies as it has a higher clindamycin MIC and increased virulence factor expression, compared to the *emm92*-15 strain^30,31^. Following incubation in antibiotic free media for 24 hours, the epidermal wound was infected with the *emm92*-3 strain (3.3 × 10^6^-1.1 × 10^7^ CFUs) (Fig. 4a), as before^32^.

Skin equivalents were imaged on day 1 and day 6 to assess group differences in wound closure and histology. Here, closure of the punch biopsy site, indicative of epidermal regrowth over time, was seen in both untreated (media) and GW3965-treated non-infected controls, as well as in infected wounds treated with GW3965. In contrast, the punch biopsy wound remained visible by day 6 for the untreated *emm92*-infected tissues (Fig. 4b). H&E- and Gram-staining of tissues on day 1 (24-hours post-infection) indicated the presence of *emm92* microcolonies (*e.g*., initial colonization) along the wound bed and minimal keratinocyte regrowth, whereas re-epithelialization around the wound perimeter was observed in the non-infected media control specimens (Fig. 4c, d). The remaining tissues were treated daily for 5 days from the bottom chamber using either media supplemented with 12.5 μM GW3965 (two times the MIC) or drug-free culture media. As expected, epidermal wound closure was observed in both non-infected controls (e.g., GW3965-treated and non-treated) by the day 6 endpoint (Fig. 4e, f). This was in direct contrast to non-GW3965-treated wounds infected with *emm92* iGAS, which uniformly demonstrated incomplete wound closure, and delayed keratinocyte re-epithelization with bacterial microcolonies invading deeply into tissues (Fig. 4g, i). Importantly, wounds infected with *emm92* iGAS that were treated with GW3965 exhibited complete or near complete epidermal recovery with only minimal bacteria observed superficially in tissues (Fig. 4h, i). Altogether, our data suggests that treatment with GW3965 in this model: (i) is non-toxic to human cultured cells, (ii) promotes wound closure in infected tissues, and (iii) supports bacterial clearance in the wound bed and dermis.

### GW3965 aids infection recovery in a murine SSTI model

We first established the skin and soft tissue infection (SSTI) model using SKH1 mice for the WV-iGAS *emm92* strain based on our previous methods employed for *emm1, emm3, emm28*, and *emm41* strains^32,33^, and existing data in CD1 mice^34^. To determine an effective inoculum dose, we subcutaneously injected hairless immunocompetent male SKH1 mice with a 10^7^-10^9^ inoculum range of the *emm92*-3 strain. Of doses evaluated, the 10^9^ inoculum was selected for all subsequent studies due to it eliciting consistent lesion development, and a significant 10% decrease in the initial body weight (Fig. S2a). At the evaluated inoculums *emm92* infection did not lead to significant changes in body temperature (Fig. S2b). In an extended 24-day study, we assessed the general infection course for the *emm92* strain using measurements of gross skin pathology and body weight change (Fig. S2c-e). Skin lesions developed in mice by 24–48 hours post-infection and self-resolved in 55% of mice by day 24 (Fig. S2c). Colony counts from skin lesions on day 24 demonstrated sterility in 81% of mice. Additionally, GAS was not detected in either the blood or spleen. As illustrated by representative images (Fig. S2e) the natural course of gross pathological findings included a progression from visible and palpable dermal abscess formation (day 2) to epidermal ulceration (day 10), and finally to healed, scarred lesions (day 22).

We next assessed the safety of GW3965 by intraperitoneal (i.p.) delivery of 10, 30, or 50 mg/kg doses to non-infected mice (male and female) for 4 consecutive days. Compared to mice that received saline, GW3965 administration exhibited no significant impact on body weight or temperature for any of the concentrations tested (Fig. S3a, b). Existing pharmacokinetic data for GW3965 in C57BL/6 mice suggests that the compound is cleared from plasma and relevant tissues within one day following oral delivery in food^35^. Here, we determined the GW3965 concentration in SKH1 mouse plasma following i.p. delivery at 2-, 4-, and 24-hours post-injection, confirming clearance by 24 hours (Fig. S3c, d).

Based on these results, we evaluated GW3965 treatment efficacy in the SSTI model using 5 daily i.p. injections of 10 mg/kg or 30 mg/kg doses. Additional control groups included infected mice treated i.p. with 50 mg/kg of penicillin, saline, or vehicle (DMSO in saline) as well as a non-infected vehicle only group. To simulate therapeutic intervention of clinical infection, treatment was initiated one day post-infection by the *emm92* strain, with gross pathology and weight change observed daily for 18 days (Fig. 5a). Notably, the reduction in lesion area for the GW3965- (30 mg/kg) and penicillin-treated groups was indistinguishable (*p* = 0.9578), while penicillin (*p* = 0.001) and GW3965 (*p* = 0.0002) both significantly reduced lesion area compared to the vehicle infected control (Fig. 5b). Infection led to an initial 5–10% weight loss across all infected groups, with weight changing differently over time, yet all groups showed similar rates of weight gain beyond 12 days post-infection. Compared to the vehicle-treated mice, penicillin treated mice regained weight significantly faster (*p* = 0.0360) over the course of the experiment (Fig. 5c). Although no such difference was observed for the 30 mg/kg GW3965 treatment group, we did note variations in the return to baseline body weight, which occurred by day 6 for penicillin treated mice, by day 8 for the GW3965 group, and by day 11 for the vehicle control group (Fig. 5c).

Corresponding data obtained in parallel experiments from mice treated with 10 mg/kg of GW3965 indicated that a lower concentration was not sufficient to significantly reduce disease parameters (Fig. S4a-f). Altogether, a 30 mg/kg GW3965 dose significantly reduced disease severity in infected mice as measured by reduced lesion size and accelerated weight gain (Fig. 5b, c).

The presence and quantity of viable bacteria were determined for spleen, blood, and skin lesions of animals at treatment endpoint (day 6) and experiment endpoint (day 18). In this model, systemic dissemination of iGAS to the blood or spleen was not detected at any time point; also, spleen weight did not differ significantly at either timepoint (Fig. S4g, h). On day 1 (24 hours following s.c. infection with 1.8 to 5.2 × 10^9^ CFUs per mouse), recovery of 1 to 5 × 10^8^ CFUs per gram of tissue verified stable infection prior to treatment initiation (Fig. 5d). By day 6, 67% of penicillin treated mice had complete clearance (i.e. sterility) of their cutaneous wound, while the remaining mice demonstrated an average 2-log-fold reduction in bacterial burden (*p* = 0.0537). Likewise, mice treated with 30 mg/kg of GW3965 had an average 2-log-fold reduction in bacterial wound burden (*p* = 0.054). Bacterial burden for the vehicle control group decreased by 1-log (*p* = 0.07), indicating a natural course of infection clearance. At the 18-day endpoint, gross examination of skin pathology indicated that ulceration persisted in 56% of vehicle treated mice, whereas an intact and re-epithelialized epidermis was observed in 90% of penicillin treated and 70% of GW3965 treated groups (Fig. 5f). Differences in bacterial CFUs recovered on day 18 did not reach statistical significance between groups (Fig. 5e); however, in support of gross pathology findings, 70% of the mice treated with either GW3965 or penicillin had cleared iGAS infection altogether, while only 56% of mice naturally recovered from iGAS infection in the vehicle-treated group (Fig. 5g). Taken together, decreased cutaneous lesion size with more rapid reduction and overall clearance of bacterial infection, combined with faster weight gain supports the predicted antimicrobial effect of GW3965 and suggests clinical efficacy in reducing infection severity by accelerating disease recovery.

## Discussion

For iGAS infections, an increase in MLS_B_ resistance rates over the last decade is a concerning threat that challenges the efficacy of standard treatment^3^. While GAS remains susceptible to β-lactams, mutations in PBP proteins that decrease penicillin susceptibility have recently been reported^36,37^. Furthermore, extremely high bacterial burdens typically encountered in iGAS infections may limit the efficacy of peptidoglycan synthesis inhibitors via the Eagle effect^38^. As a result, combination therapy with clindamycin has become the standard treatment regimen, with demonstrated improvement in clinical outcomes likely due to reduced expression of GAS toxins and virulence factors^39,40^. For iGAS infections caused by clindamycin resistant strains, a shift to β-lactam and linezolid combination therapy has been proposed, although clinical data supporting such a change is limited^41^. The acquisition of aminoglycoside and tetracycline resistance in several iGAS strains^14–17^, including the emergent *emm92* strain, further reduces therapeutic options. To address the growing antibiotic resistance of iGAS, we sought to identify novel antimicrobials using the time- and cost-saving capabilities associated with application of machine learning and AI^42,43^.

Here, we developed a machine learning model trained to identify inhibitory molecular structures based on a screening dataset of the *emm92* growth response to a diverse set of chemical compounds. The end goal was to identify chemicals that could complement current β-lactam therapy. We found that our model could fairly predict inhibitory activity based on a compound’s SMILES. In addition to predicting well whether a compound was inhibitive at specific timepoints, our model had some predictive power to its kinetics. This model can be used to infer a chemical’s bacteriostatic or bactericidal potential, which may provide clues about possible mechanisms of action for further investigation. Within our training dataset, we identified unconventional growth-inhibiting compounds with functions like antidepressants and antipsychotics. Antidepressants have previously been investigated for antimicrobial properties and have demonstrated effects such as efflux pump inhibition, membrane disruption, and gut microbiota disturbance, notably against Gram-positive bacteria and fungi^44,45^.

Using the trained model to screen FDA-approved and investigated drugs, we identified the liver X receptor (LXR) agonist GW3965 and the leukotriene receptor antagonist montelukast, as the top two candidates with antimicrobial activity *in vitro*. As is the case for GW3965, selection of compounds known to promote the host response to infection while providing direct-antibacterial effects would be ideal. While we chose to focus on FDA-evaluated drugs like montelukast and GW3965 in this study, there is further potential to screen larger libraries of small-compounds for discovery of uncharacterized antibacterial candidates. This work demonstrates that machine learning is capable of circumventing the common hurdles of limited time and resources^42^, providing a decent model for antibiotic discovery that can be expanded upon.

Here, we evaluated whether the experimental LXR agonist GW3965 could be re-purposed as an antimicrobial compound for iGAS. Our data shows that GW3965 has a bactericidal effect *in vitro* against several globally predominant iGAS strains (i.e., *emm1* and *emm3*), as well as reduces Gram-positive staining for skin equivalents, and has an antimicrobial effect *in vivo* for the treated WV-iGAS test strain. While the use of GW3965 as an antimicrobial against GAS is novel, previous studies showed its anti-viral effect on new castle disease virus^46^ and an anti-bacterial effect against *Staphylococcus aureus* and *Listeria monocytogenes*^47^. In *S. aureus*, GW3965 inhibits growth through interaction with the FeoB iron transport protein^47^. Since GAS lacks a FeoB ortholog, the mechanism of action for GW3965 against GAS remains to be elucidated, but from our AI-assisted prediction we suspect its activity is attributed to the chlorobenzene ring of the molecule. Further medicinal chemistry approaches will explore chemical modifications to reduce its MIC and improve its efficacy for active infections. Additional optimization of the dosage, administration schedule, and delivery route may also improve the bactericidal effect of GW3965 for iGAS infection *in vivo*.

Like GW3965, montelukast was also identified to have an antimicrobial effect *in vitro*. Montelukast has previously been reported as effective for treatment of *Streptococcus pneumoniae* otitis media in a rat model^48^. Other studies have used this drug successfully in combination treatment regimens, for *Mycoplasma pneumoniae*^49^, for acute otitis media^50^, and in sepsis infections in children/infants^51^.

Montelukast is the 14th most prescribed asthma/allergy medicine in the U.S. and functions as a leukotriene receptor antagonist, blocking leukotriene D4 action on the CysLT1 receptor within the lungs/airways^52^. Despite that, montelukast has an FDA’s black box warning for neuropsychiatric effects^25,26^. Because of this warning, we decided to focus on GW3965 due to its enhanced safety profile and the limited studies in murine infection models.

GW3965 has previously been evaluated in mouse models of atherosclerosis^35^, glioblastoma^53^, diabetes^54^, and sepsis^55^ with results supporting positive effects on disease-specific parameters. However, preclinical studies have shown that prolonged use of LXR agonists may promote cardiometabolic disease (elevated triglycerides) and contribute to atherosclerosis^35,56–58^. Based on our data, we reason that short-term use of GW3965 may reduce the potential dangers of prolonged increases in plasma triglycerides, while allowing for its antimicrobial applications. In support, 5 doses of GW3965 at a 30 mg/kg concentration *in vivo* lead to a significant reduction in lesion area following treatment completion. The decrease in lesion area was prolonged after treatment was stopped in mice that were followed for 18 days, supporting the idea that even short-term treatment can reduce infection severity. Evidence from the literature supports that GW3965 can shift macrophages to a reparative state^55^, while enhancing cellular debris clearance and tissue regeneration of the nervous system^53^. Likewise, our results demonstrated that GW3965 promoted wound closure indicating enhanced tissue repair, although this effect has not been previously documented in the context of skin infection. Overall, this work contributes to growing data that supports the antimicrobial potential of GW3965 with the added benefit of aiding wound recovery.

In this study, the *emm92* strain was selected for screening use due to its antimicrobial resistance profile and its growing clinical relevance in the United States since 2010, notably in surveyed areas like West Virginia^9,13–16,19,30,59,60^. Acquired plasmid borne MLS_B_ resistance in the *emm92* background has been associated with increased expression of cytolytic and immunomodulatory iGAS virulence factors^31,34^, further compounding its growing importance. Thus, in addition to identifying novel antimicrobials against iGAS, we aimed to further characterize the *emm92* infection model using human skin equivalents *in vitro* and murine *in vivo* models. In the *in vivo* model, disease presented as an abscess that typically ulcerated within 3 days, followed by a slow reduction in lesion size and skin healing over 18–24 days. While our previous studies of *emm1* and *emm3* in this murine SSTI model lead to high mortality overall, the *emm92* strain produced a longer non-lethal infection course. In support translationally, West Virginia mortality rates associated with *emm92* infection (based on known patient data) were low in the non-IVDU (8%) and IVDU populations (3%), with the majority of infections presenting as SSTIs of the limbs. Therefore, our previous patient data^15,16^ and the infection course observed in the mouse model indicates that the emergent *emm92* strain specializes in long-term survival in the wound rather than widespread dissemination. Likewise, drug therapies should address this shift by promoting bacterial killing and stimulating wound healing.

Altogether, we have shown GW3965 to be a potentially beneficial therapeutic agent against an emerging antimicrobial strain of iGAS in several models. Our investigation determined that GW3965 (i) demonstrated bactericidal activity, (ii) produced no apparent short-term adverse effects in a *in vivo* murine model, and (iii) promoted wound closure in both an *in vitro* skin organoid model, as well as in a murine *in vivo* SSTI model, all of which support its potential for use in combination therapy to expedite wound recovery in conjunction with standard treatment. Further studies are needed to optimize GW3965 treatment to its full potential, but it could be added to the arsenal against growing antimicrobial threats.

## Methods

### Bacterial strains

The WV-iGAS *emm92*-15 isolate, displaying MLS_B_, aminoglycoside and tetracycline resistance was collected from an antecubital fossa abscess and identified at the J.W. Ruby Memorial hospital clinical laboratory in Morgantown, West Virginia^15^. It was used in screening assays employed to develop the machine learning dataset. The WV-iGAS *emm92*-3 strain, isolated from a mediastinal abscess identified at the J.W. Ruby Memorial hospital clinical laboratory in Morgantown, West Virginia^15^, was transformed with the pSB027 GFP-expressing plasmid containing a chloramphenicol resistance marker, as described previously^61^ to be used in skin equivalents and mouse infection models. The *emm92*-3 strain is equally resistant to MLS_B,_ aminoglycoside, and tetracycline antibiotics, and has no difference in growth or MIC when exposed to GW3965 as compared to the *emm92*-15 strain (Fig. S5). In addition, the *emm92*-3 strain has a constitutive MLS_B_ sub-phenotype which was associated with a higher clindamycin MIC and increased virulence factor expression, making this isolate an better candidate for efficacy tests in our skin equivalent and murine models^15,30,62^. Lastly, the predicted compound was also tested for growth inhibition of the MGAS315 (*emm3*) and MGAS5005 (*emm1*) isolates representing historically predominant causes of iGAS disease globally. The MGAS315 isolate was obtained in the 1980s from a patient with streptococcal toxic shock syndrome^63^, whereas MGAS5005 was obtained from the cerebrospinal fluid of a patient in 1996^64^. The *Lactococcus lactis* strain MG1363^61^ was used in this study to represent a probiotic organism. All iGAS agar and broth cultures were routinely incubated at 37°C in 5% CO_2_. Overnight cultures of the WV-iGAS *emm92*-15 strain on brain heart infusion (BHI) agar were suspended in Todd-Hewitt broth supplemented with 0.2% yeast extract (THY medium) to an OD_600nm_ of 0.05 for starting cultures. Growth optimization tests performed in 48-well plates provided reproducible susceptibility outputs for iGAS growth while remaining high-throughput. All studies were completed with three experimental and biological replicates unless stated otherwise.

### Small compound library screening

To develop the machine learning model, a diverse library of 2,560 small compounds from Spectrum Library Micro Source Discovery was used to screen for growth inhibition. Compounds in the Spectrum Library Micro Source Discovery screening library were originally dissolved in 100% DMSO and stored at −20°C. DMSO can have an antimicrobial effect^65^, therefore we confirmed that a 1.25%−5% concentration of DMSO did not affect bacterial growth nor viability (Fig. S6a, b). Starting cultures were added to 48-well plates containing each compound at a 50 μM final concentration in 2.5% DMSO. Bacterial growth was measured as the OD_600nm_ value at 2, 4, 6, and 24-hour timepoints. Compounds were classified as inhibitory at and above 80% growth inhibition compared to the bacterial growth in control wells with the DMSO and saline vehicle and separated into functional groups (Table S1).

### Generation of a machine learning model and prediction of inhibitory compound structures

From the library screening dataset, a directed-message passing neural network was used to ascertain the compound structures and substructures associated with inhibition, as described previously^20^. Briefly, each compound’s chemical structure is generated from its SMILES (Simplified Molecular Input Line Entry System) string using RDKit and represented as a graph, with atoms as nodes and bonds as edges^66^. Atoms and bonds were also encoded with features, such as formal charge, chirality, and stereochemistry, that were used to inform the model of the local chemistry of neighboring bonds. Through a series of message passing steps, embedding of a dense vector representation of the entire molecule was subsequently fed through a feed-forward neural network to predict the properties of a molecule. In this model the input was a binary label indicating the effect of compounds on bacterial growth according to the categories of 0–0 (no inhibition), 1 − 0 (temporary inhibition: inhibitory within 2–6 hours), 0–1 (delayed inhibition: non-inhibitory at early timepoints but inhibitory at 24 hours), or 1–1 (complete inhibition across all timepoints). Furthermore, we employed RationaleRL to identify molecular substructures that are critical for growth inhibition in compounds predicted as active. Specifically, a Monte Carlo Tree Search explores possible substructures of each compound and selects those that retain the highest predicted activity^67^. The SMILES of predicted inhibitory substructures were then used to screen *in silico* libraries.

### In silico screening of Drug Repurposing Hub library

We used the trained model to predict inhibitory structures with antimicrobial potential from an FDA-evaluated Drug Repurposing Hub of 6,111 small molecules *in silico*. An FDA-evaluated library was selected for *in silico* screening to identify compounds with known safety profiles that could be re-purposed as antimicrobials. The output was a predictive score for inhibition between 0 (no inhibition) and 1 (full inhibition), where 1 was indicative of 80% or greater growth inhibition. A list of the top 200 compounds with predicted inhibitory activity was generated. The screened compounds were filtered by the predicted inhibitory scores, known mechanism of action, and solubility. Based on the filtration criteria, nine candidates were selected for further investigation to establish minimum inhibitory concentrations (MIC); whereas known antimicrobials were excluded.

### In vitro validation of predicted compounds

The top nine predicted compounds (montelukast, GSK461364, AMG510, siponimod, ABT-263, miltefosine, CFTR inhibitor-172, GW3965, and probucol) were purchased from MedChemExpress (Monmouth Junction, NJ) and tested for growth inhibition of the *emm92-*15 isolate *in vitro*, essentially as described for the compound library screening assays. Briefly, overnight cultures on BHI agar were used to inoculate THY medium. Starting cultures of iGAS at an OD_600nm_ of 0.05 and two-fold dilutions of drugs in a concentration range of 1.56–50 μM were combined in wells. Bacterial growth was measured spectrophotometrically at OD_600nm_ over a 24-hour period in triplicate wells.

For the remaining *in vitro* and *in vivo* experiments, montelukast sodium salt and GW3965 hydrochloride were purchased from Cayman chemical (Ann Arbor, MI). To determine the effect of each compound on bacterial survival starting cultures were grown with 6.25 μM of montelukast or GW3965, as before. At the 24-hour timepoint, bacteria from wells treated with GW3965 or montelukast were plated on blood agar to determine CFU/mL. For comparison of viability, untreated and vehicle treated bacteria were also plated. The percentage of survival was calculated according to the proportion of viable bacteria from the untreated (0 μM) versus montelukast or GW3965 exposed samples. For subsequent GW3965 testing against the MGAS5005, MGAS315, and *emm92*-3 iGAS strains a concentration range of 1.56–50 μM was used to assess growth inhibition, as described above.

### GW3965 and ampicillin synergy

Checkerboard assays were used to assess synergy between GW3965 and ampicillin. As the standard of care for GAS infections are β-lactam antibiotics, we tested GW3965 with ampicillin to determine the combined effect on *emm92*-15 isolate growth. For each replicate, ampicillin and GW3965 were serially diluted 2-fold in a checkerboard pattern at concentration ranges of 0.78–12.5 μM for GW3965 and 0.0015–0.1 μg/mL for ampicillin; controls at the established MIC for each compound alone were included. The OD_600nm_ value was measured over 24 hours. The MIC for each compound separately or in combination at the 24-hour timepoint was used to calculate the fractional inhibitory concentration (FIC) index, as discussed previously^68^. The FIC index was calculated as follows for compound A (GW3965) and compound B (ampicillin): FIC index = FIC_A_ + FIC_B_ = A/MIC_A_ + B/MIC_B_. FIC-values of < 0.5, > 4, or 0.5–4 were considered as synergistic, antagonistic, or indifferent effects, respectively.

### Human skin equivalent GAS infection model

Wounded full-thickness EpiDerm-FT (MatTek, Boston, MA) *in vitro* cultured human skin equivalents were used to test GW3965 efficacy. Tissue discs with an 8 mm diameter are provided in transwells, with a 3-mm wound made in the center by punch biopsy of the keratinocyte layer. Wounds were inoculated with 10 μL of the GFP-expressing log-phase *emm92*-3 strain on Day 0, resulting in infection with 3.3 × 10^6^ – 1.1 × 10^7^ CFU/per tissue. To confirm effective wound establishment by GAS, infected tissues were harvested for histopathology at 24 hours post-infection (p.i.) (Day 1, no GW3965 treatment), or after 6 days to assess baseline wound healing. Tissues were incubated in a humid environment at 37°C in 5% CO_2_ with daily-fresh media; with duplicate tissues per condition. Beginning on Day 1, tissues were treated with 12.5 μM of GW3965 delivered daily in culture media placed in the bottom chamber of the transwells. At end points half of each tissue was fixed with 10% formalin for histopathological evaluation. Scanned histology slides were viewed in a blinded fashion and annotated using SlideVault software (Huron Digital Pathology, Ontario, Canada).

### Animal use and welfare

All experiments were performed in 5-week-old male and female, immunocompetent, outbred, hairless mice (strain Crl:SKH1-hrBR) (Charles River, Wilmington, MA). Animal experiments were conducted in compliance with the regulations and standards under the Animal Welfare Act, the Public Health Service Policy on Humane Care and Use of Laboratory Animals, and the Guide for the Care and Use of Laboratory Animals. The protocol 1602000144_R2 was approved by the West Virginia University Institutional Animal Care and Use Committee (IACUC).

### Skin and soft tissue (SSTI) mouse model of iGAS infection

A range of inoculum were initially tested to establish an effective infection dose with the *emm92* iGAS bacteria in the murine SSTI model. Mice were anesthetized via isoflurane inhalation (3% in oxygen) and injected subcutaneously (s.c.) in the right flank with 10^7^, 10^8^, or 10^9^ CFU of *emm92*-3 iGAS, as described previously^33^. Gross pathophysiology of infection was measured based on whether a lesion developed, changes in mouse weight, and effect on external body temperature for the first 4 days and then every 2 days for the remainder of the 11 days of experiment.

An inoculum size of ~ 1 × 10^9^ CFU/per mouse was established as optimal based on consistent production of skin pathology with resolution over the length of 24-day experiments. The percentage of weight change overtime and lesion size were measured daily for 24 days. Abscess dimensions (length [L] and width [W]) for each mouse were recorded daily to calculate area [A = p (L/2) × (W/2)] and volume [V = 4/3p(L/2)^2^ × (W/2)] with equations for a spherical ellipsoid^33^.

### GW3965 toxicity and plasma pharmacokinetics

Groups of 2 mice were intraperitoneally (i.p.) injected with 10, 30, or 50, mg/kg of GW3965 dissolved in 5% DMSO to assess toxicity in the SKH1 mouse model. GW3965 was administered daily for 4 days, and mice were observed for the effect of treatment on weight and temperature. Changes in weight were calculated for each individual mouse based on the percentage change as compared to baseline weight before treatment began (Day 0).

To determine plasma drug concentration, blood was collected by submandibular puncture from untreated mice in tubes coated with 0.5 M EDTA (pH 7.4). 50 mM EDTA was added to prevent coagulation, and plasma was isolated by centrifugation at 1400 g for 10 minutes. For standard curve preparation, 40 μL of blank plasma from untreated mice was spiked with 100–900 ng/mL of GW3965, followed by precipitation with 400 μL of acetonitrile. Detection of GW3965 by LC-MS was performed on the AB Sciex Exion LC model and the AB Sciex QTrap5500 mass spectrometer (AB Sciex, Framingham, Massachusetts) using a Phenomenex Luna Omega 1.6 μm C18 100Å, 50 × 2.1 mm column (Torrance, California), as described^69^. The transition for GW3965 is 583.9 > 181.2 m/z (Q1 > Q3). Analyst 1.7.2 software was used for data acquisition of LC-MS readouts. A calibration curve was used to calculate the concentration recovered from the plasma standards using MultiQuant3.0.3 software (AB Sciex, Framingham, Massachusetts). For GW3965 pharmacokinetics in mouse blood during infection treatment, mice were bled at 2, 4, and 24 hours after i.p injection of the compound. Blood was collected and processed as above, and GW3965 concentration in plasma was measured via the described LC-MS method.

### In vivo GW3965 efficacy

Mice were injected s.c. with 1.8–5.2 × 10^9^ CFU of the *emm92* strain, as above. Starting on day 1 (24 hr p.i.), a 10-mg/kg (~ 16.2 μM) or 30-mg/kg (~ 48.6 μM) dose of GW3965 was administered i.p. for 5 days. Control groups of mice received a 50-mg/kg dose of Penicillin G in saline, saline alone, or vehicle (DMSO) using the same schedule. Mice were observed for the effect of treatment on the gross pathophysiology of infection for either 6- or 18-day endpoints. Data was collected as described above. Blood and organ/tissue (e.g., spleen and skin) samples were collected for enumeration of bacterial burden at the indicated times. To assess bacterial burden, the spleens and remaining halves of skin sample were homogenized and serially diluted for plating on blood agar and BHI agar containing 8 μg/mL of erythromycin. Whole blood was plated on blood agar for detection of bacteriemia.

### Statistical analysis

GraphPad Prism software was used for statistical analysis. For *in vitro* growth assays and skin equivalent experiments statistical significance was determined by either One-Way ANOVA or Unpaired *t*-tests with Welch’s correction unless otherwise noted. Data from mouse experiments was assessed for differences between treatment groups over the course of infection using a Two-way ANOVA with Tukey’s multiple comparison or a mixed model ANOVA with Tukey’s multiple comparison, as was necessary according to missing values.

## Figures and Tables

**Table 1:** Compounds predicted *in silico*

Compound	Score	Known Mechanism ofAction	MIC
Montelukast	0.700	Cysteinyl leukotriene 1(CysLT1) receptor antagonist	**6.25 μM**
GSK461364	0.636	Kinase inhibitor	>50 μM
AMG-510	0.634	Covalent inhibitor of K-Rasg12C	>50 μM
Sipionimod	0.621	Agonist of sphingosine-1-phosphate receptor 1 (S1P1) and S1P5	>50 μM
ABT-263 (Navitoclax)	0.599	Inhibitor of the Bcl-2 family proteins	>50 μM
Miltefosine	0.597	Inhibits phosphatidylcholine biosynthesis	25 μM
CFTR inhibitor-172	0.586	Selectively blocks the CFTR channel in a voltage-independent manner	>50 μM
Probucol	0.565	Antioxidant that inhibits oxidation of LDL cholesterol	>50 μM
GW3965	0.574	Liver X receptor (LXRα and LXRβ) agonist	**6.25 μM**

## Data Availability

All data generated or analyzed during this study are included in this published article and its supplementary information files.
